# Experiments and Observations on Land and Sea Air

**Published:** 1801-10

**Authors:** Adam Seybert


### *Experiments and Observations on Land and Sea Air.* By ADAM SEYBERT, M.D.

[ Concluded from p. 208, of our laſt. ]

THAT the air at ſea ſhould appear nearly of the ſame purity in different latitudes, does by no means aſtoniſh me; for if land air has certain matters mixed with it, they are *perhaps* abſorbed; and if my ſuppoſition be true, that the influence of the ſun's rays on the water tends to increaſe its purity, the opinion I entertain is not ſurpriſing. For when once purified, there are perhaps none, or few cauſes to render the air noxious after it is wafted from our towns and cities over a large body of water.

It occurred to me that probably the purity of the air at ſea varied at different periods of the day: To ſatisfy myſelf on this point, I made ſeveral trials on the 10th and 17th of June laſt. On the 10th I performed them at 9 o'clock A. M. at 12, and at 6 o'clock P. M. On the 17th at 9 A. M. and at 12 o'clock. The reſult of all the experiments of the ſame day was exactly ſimilar, at leaſt not perceptibly different.

Whether or not ſea air might be rendered more pure by agitation with water, appeared to me to be a queſtion worthy of being aſcertained, particularly as ſome celebrated men reaſon that it has this effect, and muſt hence be looked upon as one of the the greateſt reſources which we have for purifying the atmoſphere. Sir John Pringle and Dr. Ingenhouſz are of this opinion; but ſome of Dr. Prieſtley's experiments ſeem to contradict it; and ſo does the following aſſertion of the celebrated Scheele, who ſays, "L'air ne s'unit pas avec l'eau commune." Traité de l'air et du feu, p. 51.

My experiments on this head are as follow: On the 26th and 28th of June, the 2d and 5th of July, equal bulks of ſea water and air were agitated for half an hour in my eudiometer tube, but I never diſcovered any abſorption to have taken place; neither was the air rendered purer, as was evident from a mixture with nitrous air.

It now appeared probable to me, that ſea water was already ſaturated with all the gaſeous particles it could abſorb; and that freſh water, when agitated with ſea air, might diminiſh its bulk or alter its purity. In conſequence of this ſuppoſition, equal bulks of ſea air and freſh water were agitated as above; but it was not in the leaſt altered. Not entirely ſatisfied of the fallacy of my conjecture, I boiled ſea water a ſufficient time to purge it of the air it might contain; I then agitated ſea air with this boiled water, as abovementioned, and found no difference in reſult from the other experiments. Theſe reſults tend to confirm me in my belief, that if ſea water purifies the air, it is rather by adding a ſomewhat, than by abſorbing any conſiderable quantity of effluvia floating therein. Though by this I do not mean to ſay, that certain matters foreign to our atmoſphere do not float therein on land; if they exiſt, perhaps they may by water be ſubject to abſorption.

